# Absence of high-risk HPV 16 and 18 in Chinese patients with oral squamous cell carcinoma and oral potentially malignant disorders

**DOI:** 10.1186/s12985-016-0526-2

**Published:** 2016-05-20

**Authors:** Xiao-Jie Chen, Kai Sun, Wei-Wen Jiang

**Affiliations:** Department of Oral Mucosal Diseases, Shanghai Ninth People’s Hospital, Shanghai Jiao Tong University School of Medicine, New Out-patient Building, 500 Quxi Road, Shanghai, 200011 China

**Keywords:** HPV16, HPV18, Oral squamous cell carcinoma, Oral potentially malignant disorders

## Abstract

**Background:**

The critical role of human papillomavirus (HPV) in cancer has been recognized, but the involvement of HPV in oral squamous cell carcinoma (OSCC) and oral potentially malignant disorders (OPMD) is still controversial. The aim of this study was to identify and verify the prevalence of high-risk HPV infection (HPV16 and 18) in Chinese patients with OSCC or OPMD using real-time PCR and DNA sequencing.

**Methods:**

Paired tissue and serum DNA samples were extracted from 40 Chinese patients with OSCC and 59 with OPMD. A SYBR Green-based real-time PCR assay was developed to detect the E6 gene of HPV16 and HPV18. Suspicious positive samples were then sequenced to eliminate false positives.

**Results:**

We found that none of the tissue and serum samples of OSCCs and OPMDs were positive for HPV16 E6 or 18 E6, using both real-time PCR and DNA sequencing. Overall, 3 of 198 (1.52 %) and 7 of 198 (3.54 %) samples were false-positive for HPV16 E6 and HPV18 E6, respectively, using real-time PCR.

**Conclusion:**

The lack of HPV16 and HPV18 detected in this study indicates that high-risk HPV 16 and 18 infections are uncommon in Chinese patients with OSCC and OPMD. Real-time PCR followed by DNA sequencing for HPV DNA detection is an effective strategy to rule out false positives.

## Background

Head and neck squamous cell carcinoma (HNSCC), which includes squamous cell carcinomas of the oral cavity, oropharynx, larynx, and hypopharynx, is the sixth most common cancer worldwide [1, 2]. Two main risk factors related to HNSCC are tobacco use and alcohol consumption [3]. Recently, investigators have suggested that human papillomavirus (HPV) is a potential etiological factor of HNSCC in patients who do not smoke or drink alcohol, particularly in oropharynx squamous cell carcinoma (OPSCC) [4, 5]. The oncogenic proteins E6 and E7 of high-risk HPVs, such as HPV16 and HPV18, are considered to be associated with the carcinogenic process of OPSCC by inactivating the tumor suppressor genes p53 and Rb [6, 7]. However, the rate of detecting HPV in OSCC varies widely (0–100 %), and the role of HPV in oral carcinogenesis has long been controversial [8].

HPV has been detected in not only cervical cancer but in cervical premalignant lesions as well, and the detection rate is known to increase with the severity of disease abnormality [9]. Oral lesions and conditions associated with a risk of malignant transformation have been referred to as oral potentially malignant disorders (OPMD) and include oral leukoplakia (OLK), lichen planus, and erythroplakia [10]. Recent studies have revealed a varying rate of detected HPV in OPMD [8]. A better understanding of the true presence of HPV in OSCC and OPMD may thus contribute to further studies of these diseases.

Different techniques have been used to detect HPV, including in situ hybridization (ISH), Southern blot hybridization, dot blot hybridization, hybrid Capture 2 (hc2), conventional PCR, and real-time PCR [11]. ISH, Southern blot and dot blot hybridization are time-consuming procedures that require relatively large amounts of purified DNA [11]. Hc2 assay cannot genotype single HPV subtypes [11]. Of these methods, studies using PCR techniques have reported a higher sensitivity for HPV detection [12]. However, conventional PCR assays may have a lower sensitivity and specificity [11]. Real-time PCR has a sensitivity of 92 % and a specificity of 97 % in detecting HPV and is able to genotype and quantitate HPV viral load [13].

The aim of our study was to identify the detection rate of high-risk HPV types 16 and 18 in Chinese patients with OSCC and OPMD using real-time PCR and DNA sequencing.

## Methods

### Subjects

A total of 99 patients including 40 OSCC and 59 OPMD patients were enrolled from the Department of Oral Mucosal Diseases and the Department of Oral Maxillofacial Surgery at the Shanghai 9th People’s Hospital, Shanghai Jiao Tong University School of Medicine. Paired tissue and serum samples were collected from each patient. Tissue samples were immediately frozen at −80 °C after surgery. Serum was obtained from the supernatant of the collected whole blood and stored at −80 °C until processing. Histological diagnoses were made by one pathologist who was on duty and confirmed by a superior pathologist according to the World Health Organization criteria [14, 15]. This study was approved by an Independent Ethics Committee of Shanghai Ninth People's Hospital affiliated to Shanghai Jiao Tong University, School of Medicine (#200703), and signed informed consent was obtained from each patient. The baseline characteristics of the patients are presented in Table 1.

**Table 1:** The baseline characteristic of patients

ID	Diagnosis	Age	Gender	Smoking	Alcohol	Stage TNM^a^	Notes	Tumor Site	Type
CXJ 1	OSCC	77	M	Past	Never		real-time PCR	Tongue	
CXJ 2	OLK	48	F	Never	Sometimes		real-time PCR	Gingiva	
CXJ 3	OLK	36	M	Current	Current		real-time PCR	Buccal	
CXJ 4	OSCC	63	M	Never	Past		real-time PCR	Tongue	
CXJ 5	OSCC	54	M	Current	Past		real-time PCR	Buccal	Papillary
CXJ 6	OSCC	60	M	Current	Current		real-time PCR	Buccal	
CXJ 7	OSCC	41	M	Current	Current		real-time PCR	Tongue	
CXJ 8	OSCC	53	M	Past	Current		real-time PCR	Buccal	
CXJ 9	OSCC	41	M	Current	Current		real-time PCR	Floor of mouth	
CXJ 10	OSCC	69	F	Never	Never	T1M0N0	real-time PCR & DNA sequencing (18 ZDNA)	Gingiva	
CXJ 11	OSCC	56	M	Current	Never		real-time PCR & DNA sequencing (16 SDNA)	Buccal	
CXJ 12	OSCC	60	F	Never	Never		real-time PCR	Gingiva	
CXJ 13	OSCC	58	M	Current	Sometimes		real-time PCR	Gingiva	
CXJ 14	OSCC	57	F	Never	Never	T2N0M0	real-time PCR	Tongue	
CXJ 15	OSCC	55	M	Sometimes	Sometimes		real-time PCR	Hard palate	Papillary
CXJ 16	OSCC	75	M	Never	Sometimes		real-time PCR	Buccal	
CXJ 17	OSCC	66	F	Never	Never	T4N1M0	real-time PCR	Buccal	
CXJ 18	OSCC	63	M	Never	Never	T4N0M0	real-time PCR	Buccal	
CXJ 19	OSCC	43	M	Current	Current	T4N0M0	real-time PCR	Gingiva	
CXJ 20	OLK	65	M	Never	Sometimes		real-time PCR	Hard palate	
CXJ 21	OLK	56	M	Current	Sometimes		real-time PCR	Buccal	
CXJ 22	OLK	78	F	Never	Never		real-time PCR	Tongue	
CXJ 24	OSCC	59	M	Current	Current		real-time PCR	Gingiva	
CXJ 25	OSCC	54	F	Never	Never	T1N0M0	real-time PCR	Tongue	
CXJ 26	OSCC	72	M	Never	Never	T3N0M0	real-time PCR	Tongue	
CXJ 27	OSCC	75	F	Never	Never	T1N0MO	real-time PCR	Tongue	
CXJ 28	OSCC	40	M	Sometimes	Never		real-time PCR	Gingiva	
CXJ 30	OLK	56	F	Never	Never		real-time PCR & DNA sequencing (16 SDNA)	Gingiva	
CXJ 31	OLK	60	M	Current	Never		real-time PCR	Gingiva	
CXJ 32	OSCC	44	M	Current	Current		real-time PCR	Floor of mouth	
CXJ 33	OLK	65	M	Never	Never		real-time PCR	Buccal	
CXJ 34	OSCC	81	M	Never	Never		real-time PCR	Lip	
CXJ 35	OLK	63	M	Never	Never		real-time PCR	Gingiva	
CXJ 36	OSCC	58	F	Never	Never		real-time PCR	Tongue	
CXJ 37	OLK	72	M	Never	Never		real-time PCR	Gingiva	
CXJ 38	OLK	75	M	Never	Never		real-time PCR	Buccal	
CXJ 39	OLK	73	M	Past	Never		real-time PCR	Tongue	
CXJ 40	OSCC	60	F	Never	Never		real-time PCR	Buccal	
CXJ 41	OLK	36	M	Never	Sometimes		real-time PCR	Gingiva	
CXJ 42	OLK	57	F	Never	Never		real-time PCR	Buccal	
CXJ 43	OLK	51	M	Past	Past		real-time PCR	Tongue	Verrucous
CXJ 44	OLK	54	M	Past	Never		real-time PCR	Gingiva	
CXJ 45	OLK	56	M	Never	Sometimes		real-time PCR	Tongue	
CXJ 46	OLK	66	F	Current	Never		real-time PCR	Gingiva	
CXJ 47	OLK	62	M	Never	Past		real-time PCR	Tongue	
CXJ 48	OLK	50	F	Never	Never		real-time PCR	Gingiva	
CXJ 49	OSCC	63	M	Current	Sometimes		real-time PCR	Buccal	
CXJ 50	OLK	53	F	Never	Never		real-time PCR	Tongue	
CXJ 51	OLK	54	M	Current	Past		real-time PCR	Soft palate	Verrucous
CXJ 52	OLK	30	M	Current	Sometimes		real-time PCR	Tongue	
CXJ 53	OLK	62	M	Current	Sometimes		real-time PCR	Soft palate	
CXJ 54	OLK	64	F	Never	Never		real-time PCR	Buccal	
CXJ 55	OSCC	70	M	Past	Current		real-time PCR	Buccal	
CXJ 56	OLK	50	F	Never	Never		real-time PCR & DNA sequencing (16 SDNA, 18 SDNA)	Tongue	
CXJ 57	OSCC	73	F	Never	Never		real-time PCR & DNA sequencing (18 SDNA)	Buccal	
CXJ 58	OLK	59	F	Never	Never		real-time PCR	Tongue	
CXJ 59	OLK	62	F	Never	Never		real-time PCR	Gingiva	
CXJ 60	OLK	57	F	Current	Never		real-time PCR	Tongue	
CXJ 61	OLK	51	M	Current	Never		real-time PCR	Tongue	
CXJ 62	OLK	50	F	Never	Never		real-time PCR	Tongue	
CXJ 63	OSCC	67	M	Current	Never		real-time PCR	Buccal	
CXJ 64	OLK	64	M	Never	Never		real-time PCR	Tongue	
CXJ 65	OLK	45	F	Never	Never		real-time PCR	Gingiva	
CXJ 66	OLK	60	M	Never	Never		real-time PCR	Buccal	
CXJ 67	OLK	66	F	Never	Never		real-time PCR	Tongue	
CXJ 68	OSCC	38	M	Current	Sometimes		real-time PCR	Tongue	
CXJ 69	OSCC	61	M	Past	Past		real-time PCR & DNA sequencing (18 ZDNA)	Buccal	
CXJ 70	OLK	52	F	Never	Never		real-time PCR	Tongue	
CXJ 71	OLK	35	M	Past	Sometimes		real-time PCR	Buccal	
CXJ 72	OLK	58	F	Never	Never		real-time PCR	Buccal	
CXJ 73	OLK + EK	37	M	Past	Past		real-time PCR	Tongue	
CXJ 74	OSCC	34	M	Current	Current		real-time PCR	Tongue	
CXJ 75	OSCC	53	M	Current	Current		real-time PCR	Tongue	
CXJ 76	OLK	71	F	Never	Never		real-time PCR	Tongue	
CXJ 77	OSCC	58	F	Never	Never		real-time PCR	Tongue	
CXJ 78	OLK	58	F	Never	Never		real-time PCR	Buccal	
CXJ 79	OLK + EK	37	F	Never	Never		real-time PCR	Tongue	
CXJ 80	OLK	53	M	Past	Current		real-time PCR	Tongue	
CXJ 81	OSCC	58	M	Past	Sometimes		real-time PCR	Tongue	
CXJ 82	OLK	55	F	Never	Never		real-time PCR	Tongue	
CXJ 83	OLK	53	M	Current	Current		real-time PCR	Tongue	
CXJ 84	OLK	53	M	Current	Current		real-time PCR	Hard palate	Verrucous
CXJ 85	OLK	54	F	NA	NA		real-time PCR	Tongue	
CXJ 86	OLK	54	F	Never	Never		real-time PCR	Tongue	
CXJ 87	OLK	63	M	Sometimes	Current		real-time PCR	Tongue	
CXJ 88	OLK	72	M	Never	Current		real-time PCR	Gingiva	
CXJ 89	OLK	79	F	Never	Never		real-time PCR	Buccal	
CXJ 90	OLK	55	M	Past	Sometimes		real-time PCR	Tongue	
CXJ 91	EK	45	F	Never	Never		real-time PCR	Buccal	
CXJ 94	OLP	54	F	Never	Never		real-time PCR	Buccal	
CXJ 95	OLP	54	F	Never	Never		real-time PCR & DNA sequencing (18 SDNA)	Buccal	
CXJ 96	OLP	29	M	Current	Sometimes		real-time PCR	Buccal	
CXJ 97	OLP	40	F	Never	Never		real-time PCR	Buccal	
CXJ 98	OLP	58	F	Never	Never		real-time PCR	Buccal	
CXJ 99	OLP	28	M	Current	Never		real-time PCR	Buccal	
CXJ 100	OSCC	28	M	Current	Current		real-time PCR & DNA sequencing (18 ZDNA)	Buccal	
CXJ 101	OSCC	62	F	Never	Never		real-time PCR	Gingiva	
CXJ 102	OSCC	68	M	Never	Never		real-time PCR	Tongue	
CXJ 103	OSCC	59	M	Never	Never		real-time PCR & DNA sequencing (18 ZDNA)	Buccal	

*OSCC* oral squamous cell carcinoma, *OLK* oral leukoplakia, *OLP* oral lichen planus, *EK* oral erythroplakia, *ZDNA* tissue DNA, *SDNA* serum DNA, *NA* data not available

^a^Union for International Cancer Control; T, tumor size; N, lymph node; M, Metastasis

### Cell culture

The CAL27 cell line was obtained from the American Type Culture Collection (ATCC, Rockville, MA, USA) and was grown in Dulbecco’s Modified Eagle Medium (HyClone, Logan, UT, USA) containing 10 % fetal bovine serum (FBS) and 1 % penicillin-streptomycin solution at 37 °C in 5 % CO_2_.

### DNA extraction

Twenty 20-μm sections were cut from the frozen tissue samples, and DNA was extracted using the QIAamp DNA Micro Kit (Qiagen, Düsseldorf, Germany). Serum DNA extraction was performed using the QIAamp DNA Blood Mini Kit (Qiagen, Düsseldorf, Germany). CAL27 cells were detached by trypsinization and extracted DNA with QIAamp DNA Mini Kit (Qiagen, Düsseldorf, Germany). The plasmid pB-actin 16 E6 and pB-actin 18 E6 were bought from Addgene (Cambridge, MA, USA). Plasmid DNA was extracted using the QIAfilter MidiKit (Qiagen, Düsseldorf, Germany). Purified plasmid DNA were sequenced and blasted with HPV16 E6 (NC_001526.2) and HPV18 E6 (NC_001357.1) NCBI reference sequence. The extracted DNA was stored at −80 °C until further use.

### Real-time PCR and sequencing

**Fig. 1:**
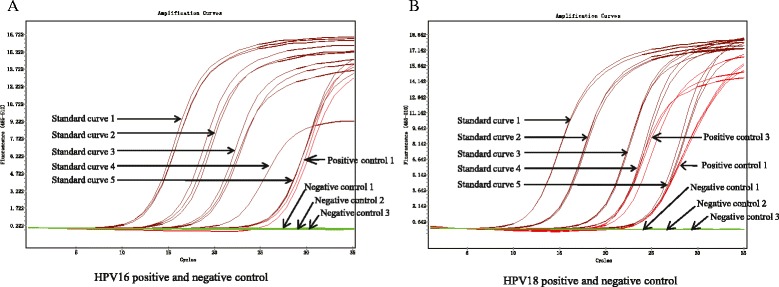
Positive and negative controls for HPV16 and HPV 18 with real-time PCR. **a** Positive and negative controls for HPV16; **b** Positive and negative controls for HPV18. Standard curve 1–5, 10-fold diluted HPV16 E6 or HPV18 E6 plasmid DNA ranging from 1 ng/well to 0.1 pg/well. Positive control 1, cilnical DNA sample added with 0.1 pg HPV16 E6 or HPV18 E6 DNA. Positive control 2, 50 ng Hela cell DNA. Negative control 1, pure water. Negative control 2, pure water instead of 2 × master mixture. Negative control 3, pure water instead of positive control DNA

**Fig. 2:**
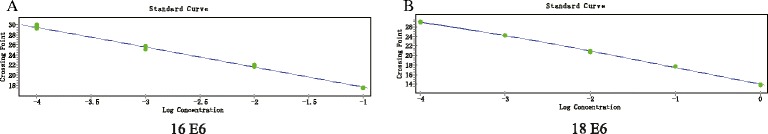
Standard curves for HPV16 and HPV 18 E6. **a** Standard curves for 10-fold diluted HPV16 E6 plasmid DNA ranging from 0.1 ng/well to 0.1 pg/well; **b** Standard curves for 10-fold diluted HPV18 E6 plasmid DNA ranging from 1 ng/well to 0.1 pg/well

Real-time PCR was performed by LightCycler 480 SYBR Green I Master (Roche, Basel, Switzerland) together with 0.5 μmol/L of each primer and 50 ng DNA in a 10 μl reaction were utilized. Positive controls were performed, which including HPV plasmid DNA, HPV containing cell line DNA and small amount of plasmids added to clinical sample DNA (Fig. 1). Negative controls were also performed, which including pure water, pure water instead of 2 × master mixture, pure water instead of positive control DNA (Fig. 1). A standard curve was developed for both HPV16 E6 (Fig. 2a) and HPV18 E6 (Fig. 2b) using a series of 10-fold diluted plasmid DNA 1 ng to 0.1 pg. The quantitated data was normalized by beta-actin (ACTB) using CAL27 genomic DNA. The reaction was performed by initiation at 95 °C for 5 min followed by 35 cycles of 95 °C for 10 s, 60 °C for 20 s and 72 °C for 10 s. Each sample was performed in triplicate. A sample was considered positive for HPV infection if two or three wells of the triplicate showed an amplifying curve. It was under suspicion if the amplifying curve was detected later than the 30^th^ cycle of the reaction or had a deformed shape. The suspicious samples of HPV16 E6 or HPV18 E6 were then sequenced to rule out false positives. All primers are shown in Table 2.

**Table 2:** Sequence of HPV16 and HPV18 E6 primers and ACTB primers used for real-time PCR

Name	Sequence
HPV16 E6-F	GTCATATACCTCACGTCGCAG
HPV16 E6-R	AGCGACCCAGAAAGTTACCAC
HPV18 E6-F	GTTTCTCTGCGTCGTTGGAG
HPV18 E6-R	GGTGCCAGAAACCGTTGAAT
ACTB-F	TCCCTCTCAGGCATGGAGTC
ACTB-R	AATGCCAGGGTACATGGTGG

## Results

**Fig. 3:**
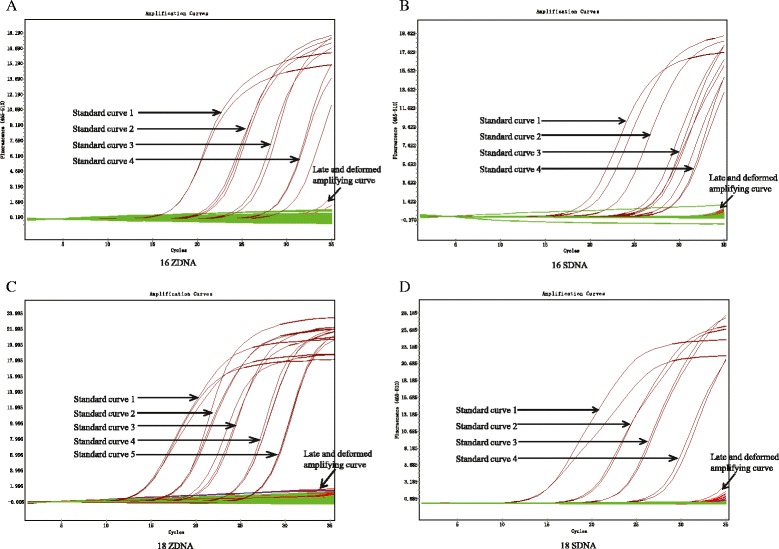
Amplification curves for HPV16 and HPV 18 with real-time PCR. **a** Detection of HPV16 E6 in tissue samples; **b** Detection of HPV16 E6 in serum samples; **c** Detection of HPV18 E6 in tissue samples; **d** Detection of HPV18 E6 in serum samples

Real-time PCR was conducted to detect HPV16 E6 and HPV18 E6 DNA. We found that zero of the 99 tissue samples (0 %) showed a standard amplifying curve for HPV 16 E6, but a few samples showed late or deformed amplifying curves in one of the triplicates, which were clearly not considered to be positive (Fig. 3a). Thirty-nine of 40 OSCC and 57 of 59 OPMD serum samples did not show a standard amplifying curve for HPV 16 E6 using real-time PCR, but 1 OSCC and 2 OPMD serum samples had a late or deformed amplifying curve in two or three wells of the triplicate that was suspicious (Fig. 3b). In addition, 36 of 40 OSCC and all 59 OPMD tissue samples were negative for the standard amplifying curve of HPV 18 E6, but 4 OSCC tissue samples presented a late and deformed amplifying curve in two or three wells of the triplicate (Fig. 3c). Thirty-nine of 40 OSCC and 57 of 59 OPMD serum samples were negative for the standard amplifying curve of HPV 18 E6, but 1 OSCC and 2 OPMD serum samples had late and deformed amplifying curves in two or three wells of the triplicate (Fig. 3d). DNA sequence analysis was then performed on the suspicious samples, which found that all of the samples sequenced were negative for HPV16 and HPV18. Overall, 3 of 198 (1.52 %) and 7 of 198 (3.54 %) samples were false-positive for HPV16 E6 and HPV18 E6, respectively, using real-time PCR. Overall, none of the OSCC or OPMD cases were positive for HPV 16 or18 in our study.

## Discussion

In the past few decades, there has been speculation worldwide about the role of HPV in the pathogenesis of HNSCC. The most commonly detected HPV, HPV16, accounts for 90 % of the HPV DNA-positive cases in HNSCC, followed by HPV18 and other high-risk subtypes [16]. However, the detection rate of HPV in OSCC and OPMD varies widely and remains controversial [8, 17]. This variation may due to differences in the types of sample, detection methods or geographic locations [8, 18]. Therefore, confirming the HPV infection rate in OSCC and OPMD cases may contribute to the study of carcinogenesis in the oral cavity [19, 20]. In this study, we used real-time PCR to detect HPV16 and HPV18 in paired tissue and serum samples of Chinese OSCC and OPMD patients [21]. We conducted complementary analyses to verify the results of the real-time PCR with DNA sequencing. We found that none of the patients with OSCC or OPMD demonstrated existence of high-risk HPV16 or HPV18. The absence of HPV DNA in our sample implies that HPV infection may not be common in Chinese patients with OSCC and OPMD.

A critical step in malignant transformation is the integration of high-risk HPV DNA into the human cellular genome, followed by the expression of the oncoproteins E6 and E7, which promote tumor progression [21]. In a previous study, although the reported detection rate of high-risk HPV DNA in OSCC was 6.6 %, HPV mRNA was only detected in 5.9 % [22]. These findings indicated that the mRNA or oncoproteins of HPV E6 and E7 were less commonly found than the DNA, as the presence of HPV in the genome differed from the HPV-related etiology [23, 24]. The gold standard to identify the presence of HPV was therefore suggested to be detecting HPV DNA [25].

Yadav et al. showed that the HPV DNA detection limit for conventional PCR was 200 copies, whereas for real-time PCR, which has a higher sensitivity, detecting HPV DNA required only 1 copy [26]. Lingen et al. detected high-risk HPV DNA in 9.8 % of OSCC cases using consensus primer PCR, but the positive rate was 6.6 % using real-time PCR [22]. Scapoli et al. found the detection rate of HPV16 to be 2 % in OSCC with real-time PCR [27]. Real-time PCR shows a higher sensitivity and specificity than conventional PCR assays [12, 22, 26]. In the current study, we utilized real-time PCR and found that 3 of 198 samples showed late and deformed amplifying curves of HPV 16 E6 and 7 of 198 samples had late and deformed amplifying curves of HPV 18 E6. To rule out false positives, we performed subsequent sequencing and found that the rate of false positives using real-time PCR to detect HPV16 E6 and HPV18 E6 DNA was 1.52 and 3.54 %, respectively. Ha et al. found a 2 % false-positive rate for real-time PCR using the minimum criteria of HPV DNA copy number, which was similar to our results [12].

The population has also been considered to be another factor affecting rate diversification. Several countries have revealed a zero detection rate of OSCC, including India [28–30], Brazil [31], Japan [32] and Mozambique [33]. Other reported detection rates have been 1.54 % in Thailand [34], 6.6 % in America [22], 5 % in Mexico [35], 39.4 % in Spain [36] and 66.7 % in Sudan [37]. Studies performed in China have yielded varied results using conventional PCR assays, ranging from 2.2 to 74 % [38–42]. However, real-time PCR data for OSCC has not been reported in China. Our study revealed a zero detection rate of HPV16 and 18 in OSCC by combining real-time PCR and DNA sequencing, which was a reliable method and provided further understanding of HPV infection in Chinese patients.

HPV infection has been identified in cancers of the cervix [43], vulva [44], vagina [44], anus [44], penis [45] and oropharynx [46]. It is widely accepted that OPSCCs, especially tonsillar cancers, are frequently associated with HPV infection [17]. The recent reported prevalence of HPV in OPSCC was approximately 60-70 % [47], but the corresponding rate was substantially lower and significantly varied in OSCC [8, 17]. HPV prevalence in OPSCC has been suggested to be an independent prognostic factor [47]. HPV-positive OPSCC has been shown to be distinct from HPV-negative OPSCC with regard to prognosis [48–50]. However, there have been no direct correlations between HPV infection and oral carcinogenesis [23, 27, 51].

HPV has been detected not only in cancer but also in premalignant lesions, such as in lesions of the cervix and breast [9, 52, 53]. In contrast, there was a lack of HPV in premalignant lesions of the colon [54–56]. Interestingly, Ha et al. demonstrated a low prevalence (1.1 %) of HPV16 in OPMD [12]. Similarly, we detected no presence of HPV 16 and 18 in Chinese patients with OPMD.

## Conclusion

Overall, we demonstrated a prevalence rate of 0 % of HPV 16 and 18 in Chinese patients with OSCC and OPMD. Our data suggests that high-risk HPV16 and HPV18 infection may not be common in Chinese patients with OSCC and OPMD. Combining real-time PCR and DNA sequence for HPV DNA detection is an effective strategy to eliminate false positives.

## Abbreviations

HNSCC: head and neck squamous cell carcinoma
HPV: human papillomavirus
OPSCC: oropharynx squamous cell carcinoma
OSCC: oral squamous cell carcinoma

## References

[1] van Kempen PM, Noorlag R, Braunius WW, Moelans CB, Rifi W, Savola S, Koole R, Grolman W, van Es RJ, Willems SM. Clinical relevance of copy number profiling in oral and oropharyngeal squamous cell carcinoma. Cancer Med. 2015;4:1525–35. http://www.ncbi.nlm.nih.gov/pubmed/26194878. 10.1002/cam4.499 PMC4618623 26194878

[2] Wang Y, Springer S, Mulvey CL, Silliman N, Schaefer J, Sausen M, James N, Rettig EM, Guo T, Pickering CR, et al. Detection of somatic mutations and HPV in the saliva and plasma of patients with head and neck squamous cell carcinomas. Sci Transl Med. 2015;7:293ra104. http://www.ncbi.nlm.nih.gov/pubmed/26109104. 10.1126/scitranslmed.aaa8507 PMC4587492 26109104

[3] Badulescu F, Crisan A, Badulescu A, Schenker M (2010). Recent data about the role of human papillomavirus (HPV) in oncogenesis of head and neck cancer. Rom J Morphol Embryol.

[4] Friedman JM, Stavas MJ, Cmelak AJ (2014). Clinical and scientific impact of human papillomavirus on head and neck cancer. World J Clin Oncol.

[5] Gama RR, Carvalho AL, Filho AL, Scorsato AP, Lopez RV, Rautava J, Syrjanen S, Syrjanen K. Detection of human papillomavirus in laryngeal squamous cell carcinoma: Systematic review and meta-analysis. Laryngoscope. 2015;126:885-93. http://www.ncbi.nlm.nih.gov/pubmed/26542064. 10.1002/lary.25738 26542064

[6] Feller L, Wood NH, Khammissa RA, Lemmer J (2010). Human papillomavirus-mediated carcinogenesis and HPV-associated oral and oropharyngeal squamous cell carcinoma. Part 1: human papillomavirus-mediated carcinogenesis. Head Face Med.

[7] Feller L, Wood NH, Khammissa RA, Lemmer J (2010). Human papillomavirus-mediated carcinogenesis and HPV-associated oral and oropharyngeal squamous cell carcinoma. Part 2: Human papillomavirus associated oral and oropharyngeal squamous cell carcinoma. Head Face Med.

[8] Gupta S, Gupta S (2015). Role of human papillomavirus in oral squamous cell carcinoma and oral potentially malignant disorders: A review of the literature. Indian J Dent.

[9] Forman D, de Martel C, Lacey CJ, Soerjomataram I, Lortet-Tieulent J, Bruni L, Vignat J, Ferlay J, Bray F, Plummer M, Franceschi S. Global burden of human papillomavirus and related diseases. Vaccine. 2012;30 Suppl 5:F12–23. http://www.ncbi.nlm.nih.gov/pubmed/23199955. 10.1016/j.vaccine.2012.07.055 23199955

[10] Hassona Y, Scully C, Almangush A, Baqain Z, Sawair F (2014). Oral potentially malignant disorders among dental patients: a pilot study in Jordan. Asian Pac J Cancer Prev.

[11] Abreu AL, Souza RP, Gimenes F, Consolaro ME (2012). A review of methods for detect human Papillomavirus infection. Virol J.

[12] Ha PK, Pai SI, Westra WH, Gillison ML, Tong BC, Sidransky D, Califano JA. Real-time quantitative PCR demonstrates low prevalence of human papillomavirus type 16 in premalignant and malignant lesions of the oral cavity. Clin Cancer Res. 2002;8:1203–9. http://www.ncbi.nlm.nih.gov/pubmed/12006539. 12006539

[13] Venuti A, Paolini F (2012). HPV detection methods in head and neck cancer. Head Neck Pathol.

[14] Axell T, Pindborg JJ, Smith CJ, van der Waal I (1996). Oral white lesions with special reference to precancerous and tobacco- related lesions: conclusions of an international symposium held in Uppsala, Sweden, May 18–21 1994. International Collaborative Group on Oral White Lesions. J Oral Pathol Med.

[15] Wittekind C, Compton CC, Greene FL, Sobin LH (2002). TNM residual tumor classification revisited. Cancer.

[16] Gillison ML, Alemany L, Snijders PJ, Chaturvedi A, Steinberg BM, Schwartz S, Castellsague X. Human papillomavirus and diseases of the upper airway: head and neck cancer and respiratory papillomatosis. Vaccine. 2012;30 Suppl 5:F34–54. http://www.ncbi.nlm.nih.gov/pubmed/23199965. 10.1016/j.vaccine.2012.05.070 23199965

[17] Ha PK, Califano JA (2004). The role of human papillomavirus in oral carcinogenesis. Crit Rev Oral Biol Med.

[18] Campisi G, Panzarella V, Giuliani M, Lajolo C, Di Fede O, Falaschini S, Di Liberto C, Scully C, Lo Muzio L. Human papillomavirus: its identity and controversial role in oral oncogenesis, premalignant and malignant lesions (review). Int J Oncol. 2007;30:813–23. http://www.ncbi.nlm.nih.gov/pubmed/17332919. 17332919

[19] Chai RC, Lambie D, Verma M, Punyadeera C (2015). Current trends in the etiology and diagnosis of HPV-related head and neck cancers. Cancer Med.

[20] Sathish N, Wang X, Yuan Y (2014). Human Papillomavirus (HPV)-associated Oral Cancers and Treatment Strategies. J Dent Res.

[21] Zhao M, Rosenbaum E, Carvalho AL, Koch W, Jiang W, Sidransky D, Califano J. Feasibility of quantitative PCR-based saliva rinse screening of HPV for head and neck cancer. Int J Cancer. 2005;117:605–10. http://www.ncbi.nlm.nih.gov/pubmed/15929076. 10.1002/ijc.21216 15929076

[22] Lingen MW, Xiao W, Schmitt A, Jiang B, Pickard R, Kreinbrink P, Perez-Ordonez B, Jordan RC, Gillison ML. Low etiologic fraction for high-risk human papillomavirus in oral cavity squamous cell carcinomas. Oral Oncol. 2013;49:1–8. http://www.ncbi.nlm.nih.gov/pubmed/22841678. 10.1016/j.oraloncology.2012.07.002 22841678

[23] Yamakawa-Kakuta Y, Kawamata H, Doi Y, Fujimori T, Imai Y (2009). Does the expression of HPV16/18 E6/E7 in head and neck squamous cell carcinomas relate to their clinicopathological characteristics?. Int J Oncol.

[24] Wiest T, Schwarz E, Enders C, Flechtenmacher C, Bosch FX (2002). Involvement of intact HPV16 E6/E7 gene expression in head and neck cancers with unaltered p53 status and perturbed pRb cell cycle control. Oncogene.

[25] Zaravinos A (2014). An updated overview of HPV-associated head and neck carcinomas. Oncotarget.

[26] Yadav R, Paria A, Mankame S, Makesh M, Chaudhari A, Rajendran KV (2015). Development of SYBR Green and TaqMan quantitative real-time PCR assays for hepatopancreatic parvovirus (HPV) infecting Penaeus monodon in India. Mol Cell Probes.

[27] Scapoli L, Palmieri A, Rubini C, Martinelli M, Spinelli G, Ionna F, Carinci F. Low prevalence of human papillomavirus in squamous-cell carcinoma limited to oral cavity proper. Mod Pathol. 2009;22:366–72. http://www.ncbi.nlm.nih.gov/pubmed/18978731. 10.1038/modpathol.2008.180 18978731

[28] Laprise C, Madathil SA, Allison P, Abraham P, Raghavendran A, Shahul HP, ThekkePurakkal AS, Castonguay G, Coutlee F, Schlecht NF, et al. No role for human papillomavirus infection in oral cancers in a region in southern India. Int J Cancer. 20152015;138:912-7. http://www.ncbi.nlm.nih.gov/pubmed/26317688. 10.1002/ijc.29827 26317688

[29] Pathare SM, Gerstung M, Beerenwinkel N, Schaffer AA, Kannan S, Pai P, Pathak KA, Borges AM, Mahimkar MB. Clinicopathological and prognostic implications of genetic alterations in oral cancers. Oncol Lett. 2011;2:445–51. http://www.ncbi.nlm.nih.gov/pubmed/21546976. 10.3892/ol.2011.271 PMC3085881 21546976

[30] Patel KR, Vajaria BN, Begum R, Desai A, Patel JB, Shah FD, Shukla SN, Patel PS. Prevalence of high-risk human papillomavirus type 16 and 18 in oral and cervical cancers in population from Gujarat, West India. J Oral Pathol Med. 2014;43:293–7. http://www.ncbi.nlm.nih.gov/pubmed/24372728. 10.1111/jop.12147 24372728

[31] Rivero ER, Nunes FD (2006). HPV in oral squamous cell carcinomas of a Brazilian population: amplification by PCR. Braz Oral Res.

[32] Maruyama H, Yasui T, Ishikawa-Fujiwara T, Morii E, Yamamoto Y, Yoshii T, Takenaka Y, Nakahara S, Todo T, Hongyo T, Inohara H. Human papillomavirus and p53 mutations in head and neck squamous cell carcinoma among Japanese population. Cancer Sci. 2014;105:409–17. http://www.ncbi.nlm.nih.gov/pubmed/24521534. 10.1111/cas.12369 PMC4317800 24521534

[33] Blumberg J, Monjane L, Prasad M, Carrilho C, Judson BL (2015). Investigation of the presence of HPV related oropharyngeal and oral tongue squamous cell carcinoma in Mozambique. Cancer Epidemiol.

[34] Khovidhunkit SO, Buajeeb W, Sanguansin S, Poomsawat S, Weerapradist W (2008). Detection of human papillomavirus in oral squamous cell carcinoma, leukoplakia and lichen planus in Thai patients. Asian Pac J Cancer Prev.

[35] Gonzalez-Ramirez I, Irigoyen-Camacho ME, Ramirez-Amador V, Lizano-Soberon M, Carrillo-Garcia A, Garcia-Carranca A, Sanchez-Perez Y, Mendez-Martinez R, Granados-Garcia M, Ruiz-Godoy L, Garcia-Cuellar C. Association between age and high-risk human papilloma virus in Mexican oral cancer patients. Oral Dis. 2013;19:796–804. http://www.ncbi.nlm.nih.gov/pubmed/23379359. 10.1111/odi.12071 23379359

[36] Llamas-Martinez S, Esparza-Gomez G, Campo-Trapero J, Cancela-Rodriguez P, Bascones-Martinez A, Moreno-Lopez LA, Garcia-Nunez JA, Cerero-Lapiedra R. Genotypic determination by PCR-RFLP of human papillomavirus in normal oral mucosa, oral leukoplakia and oral squamous cell carcinoma samples in Madrid (Spain). Anticancer Res. 2008;28:3733–41. http://www.ncbi.nlm.nih.gov/pubmed/19189658. 19189658

[37] Babiker AY, Eltom FM, Abdalaziz MS, Rahmani A, Abusail S, Ahmed HG (2013). Screening for high risk human papilloma virus (HR-HPV) subtypes, among Sudanese patients with oral lesions. Int J Clin Exp Med.

[38] Tang X, Jia L, Ouyang J, Takagi M (2003). Comparative study of HPV prevalence in Japanese and North-east Chinese oral carcinoma. J Oral Pathol Med.

[39] Zhao D, Xu QG, Chen XM, Fan MW (2009). Human papillomavirus as an independent predictor in oral squamous cell cancer. Int J Oral Sci.

[40] Zhang ZY, Sdek P, Cao J, Chen WT (2004). Human papillomavirus type 16 and 18 DNA in oral squamous cell carcinoma and normal mucosa. Int J Oral Maxillofac Surg.

[41] Gan LL, Zhang H, Guo JH, Fan MW (2014). Prevalence of human papillomavirus infection in oral squamous cell carcinoma: a case-control study in Wuhan, China. Asian Pac J Cancer Prev.

[42] Chor JS, Vlantis AC, Chow TL, Fung SC, Ng FY, Lau CH, Chan AB, Ho LC, Kwong WH, Fung MN, et al. The role of human papillomavirus in head and neck squamous cell carcinoma: A case control study on a southern Chinese population. J Med Virol. 2015; 88:877-87. http://www.ncbi.nlm.nih.gov/pubmed/26467027. 10.1002/jmv.24405 26467027

[43] Guan P, Howell-Jones R, Li N, Bruni L, de Sanjose S, Franceschi S, Clifford GM. Human papillomavirus types in 115,789 HPV-positive women: a meta-analysis from cervical infection to cancer. Int J Cancer. 2012;131:2349–59. http://www.ncbi.nlm.nih.gov/pubmed/22323075. 10.1002/ijc.27485 22323075

[44] De Vuyst H, Clifford GM, Nascimento MC, Madeleine MM, Franceschi S (2009). Prevalence and type distribution of human papillomavirus in carcinoma and intraepithelial neoplasia of the vulva, vagina and anus: a meta-analysis. Int J Cancer.

[45] de Sousa ID, Vidal FC, Branco Vidal JP, de Mello GC, do Desterro Soares Brandao Nascimento M, Brito LM (2015). Prevalence of human papillomavirus in penile malignant tumors: viral genotyping and clinical aspects. BMC Urol.

[46] Singhi AD, Westra WH (2010). Comparison of human papillomavirus in situ hybridization and p16 immunohistochemistry in the detection of human papillomavirus-associated head and neck cancer based on a prospective clinical experience. Cancer.

[47] Benson E, Li R, Eisele D, Fakhry C (2014). The clinical impact of HPV tumor status upon head and neck squamous cell carcinomas. Oral Oncol.

[48] Klussmann JP, Mooren JJ, Lehnen M, Claessen SM, Stenner M, Huebbers CU, Weissenborn SJ, Wedemeyer I, Preuss SF, Straetmans JM, et al. Genetic signatures of HPV-related and unrelated oropharyngeal carcinoma and their prognostic implications. Clin Cancer Res. 2009;15:1779–86. http://www.ncbi.nlm.nih.gov/pubmed/19223504. 10.1158/1078-0432.CCR-08-1463 19223504

[49] Smeets SJ, Braakhuis BJ, Abbas S, Snijders PJ, Ylstra B, van de Wiel MA, Meijer GA, Leemans CR, Brakenhoff RH. Genome-wide DNA copy number alterations in head and neck squamous cell carcinomas with or without oncogene-expressing human papillomavirus. Oncogene. 2006;25:2558–64. http://www.ncbi.nlm.nih.gov/pubmed/16314836. 10.1038/sj.onc.1209275 16314836

[50] van Kempen PM, van Bockel L, Braunius WW, Moelans CB, van Olst M, de Jong R, Stegeman I, van Diest PJ, Grolman W, Willems SM. HPV-positive oropharyngeal squamous cell carcinoma is associated with TIMP3 and CADM1 promoter hypermethylation. Cancer Med. 2014;3:1185–96. http://www.ncbi.nlm.nih.gov/pubmed/25065733. 10.1002/cam4.313 PMC4302669 25065733

[51] Smith EM, Rubenstein LM, Haugen TH, Pawlita M, Turek LP (2012). Complex etiology underlies risk and survival in head and neck cancer human papillomavirus, tobacco, and alcohol: a case for multifactor disease. J Oncol.

[52] Manzouri L, Salehi R, Shariatpanahi S, Rezaie P (2014). Prevalence of human papilloma virus among women with breast cancer since 2005-2009 in Isfahan. Adv Biomed Res.

[53] Liang W, Wang J, Wang C, Lv Y, Gao H, Zhang K, Liu H, Feng J, Wang L, Ma R. Detection of high-risk human papillomaviruses in fresh breast cancer samples using the hybrid capture 2 assay. J Med Virol. 2013;85:2087–92. http://www.ncbi.nlm.nih.gov/pubmed/23959946. 10.1002/jmv.23703 23959946

[54] Taherian H, Tafvizi F, Fard ZT, Abdirad A (2014). Lack of association between human papillomavirus infection and colorectal cancer. Prz Gastroenterol.

[55] Aghakhani A, Hamkar R, Ramezani A, Bidari-Zerehpoosh F, Sabeti S, Ghavami N, Banifazl M, Rashidi N, Eslamifar A. Lack of human papillomavirus DNA in colon adenocarcinama and adenoma. J Cancer Res Ther. 2014;10:531–4. http://www.ncbi.nlm.nih.gov/pubmed/25313733. 10.4103/0973-1482.137674 25313733

[56] Burnett-Hartman AN, Newcomb PA, Mandelson MT, Galloway DA, Madeleine MM, Wurscher MA, Carter JJ, Makar KW, Potter JD, Schwartz SM. No evidence for human papillomavirus in the etiology of colorectal polyps. Cancer Epidemiol Biomarkers Prev. 2011;20:2288–97. http://www.ncbi.nlm.nih.gov/pubmed/21817125. 10.1158/1055-9965.EPI-11-0450 PMC3236024 21817125

